# Incidence rate and prevalence of pediatric‐onset multiple sclerosis in Sweden: A population‐based register study

**DOI:** 10.1111/ene.16253

**Published:** 2024-02-18

**Authors:** Fredrik Sandesjö, Helen Tremlett, Katharina Fink, Ruth Ann Marrie, Feng Zhu, Ronny Wickström, Kyla A. McKay

**Affiliations:** ^1^ Department of Women's and Children's Health Karolinska Institutet Stockholm Sweden; ^2^ Division of Neurology, Department of Medicine, The Djavad Mowafaghian Centre for Brain Health University of British Columbia Vancouver British Columbia Canada; ^3^ Department of Clinical Neuroscience Karolinska Institutet Stockholm Sweden; ^4^ Department of Internal Medicine Department of Community Health Sciences Max Rady College of Medicine, Rady Faculty of Health Sciences, University of Manitoba Winnipeg Manitoba Canada

**Keywords:** incidence, multiple sclerosis, pediatric, pediatric‐onset multiple sclerosis, prevalence

## Abstract

**Background and purpose:**

Pediatric‐onset multiple sclerosis (PoMS) is associated with high health care use. To plan resource allocation for this patient group, knowledge of the incidence rate and prevalence is important. However, such studies are scarce, few are population‐based, and the methodology varies widely. We aimed to address this knowledge gap by performing a nationwide study of the incidence rate and prevalence of PoMS in Sweden, an area of high multiple sclerosis (MS) incidence and prevalence.

**Methods:**

MS cases were identified by linking two nationwide registers, the National Patient Register and the Swedish MS Registry. MS cases having their first central nervous system demyelinating event or MS clinical onset before age 18 years were classified as pediatric onset. Incidence rate and prevalence were estimated annually over the study period (2006–2016) for the total population and stratified by sex and age group (<12, 12–15, and 16–17 years). Temporal trends and ratios between sexes and age groups were estimated.

**Results:**

We identified 238 incident cases from 2006 to 2016, corresponding to an overall crude incidence rate of 1.12 per 100,000 person‐years and an overall crude prevalence of 2.82 per 100,000 population. There was a higher incidence rate among females and the highest age category. The overall incidence rate and prevalence estimates remained stable during the study period.

**Conclusions:**

Sweden exhibits a consistently high incidence rate and prevalence of PoMS that has remained stable over time. This knowledge serves as a tool to aid in planning resource allocation and health services for this patient population.

## INTRODUCTION

Multiple sclerosis (MS) is a chronic, inflammatory, degenerative disease of the central nervous system [1] that affects approximately 2.8 million persons worldwide [2]. Pediatric‐onset multiple sclerosis (PoMS) accounts for a small proportion of the total MS population [3, 4] and is more inflammatory than adult‐onset MS, with a higher relapse rate [5] and faster accumulation of magnetic resonance imaging lesions [6]. The consequences of PoMS include physical and cognitive impairment, resulting in high rates of health care and disability benefits use in adulthood [7]. Awareness to ensure early recognition and appropriate treatment is therefore essential.

PoMS is rare, making it a challenging population to study; a recent review reported incidence rates ranging from 0.05 (Tunisia) to 2.85 (Sardinia) per 100,000 and prevalence estimates ranging from 0.69 (Japan) to 26.92 (Sardinia) per 100,000 persons [8]. To date, there have been no studies of the incidence or prevalence of PoMS within Sweden, an area of both high MS incidence and prevalence [9, 10]. To plan resource allocation and general health services for this patient group, knowledge of the incidence and prevalence is very important. Our study aimed to fill this knowledge gap by performing a nationwide register‐based study of the incidence and prevalence of PoMS in Sweden.

## METHODS

### Setting

Sweden is located in Northern Europe. Around 20% of Sweden's 10.5 million inhabitants are under 18 years old. Furthermore, 20% of the total population are foreign‐born. Health, medical, and dental care is divided into public and private sectors. If private health care providers are under contract with the National Healthcare Services, the cost (for the patient) of private and public health care is the same. If not, the patient must pay for the full cost of any treatment and care. In Sweden, basic health and medical care is generally called primary care. Additionally, there is specialized care requiring more focused medical measures than what is available through primary care. There is no need to have a referral from primary care before contacting specialist care [11, 12].

### Source population and data sources

MS cases were obtained from two nationwide registers, the National Patient Register and the Swedish MS Registry. The patient register contains prospectively collected health administrative data from all hospitalizations (complete coverage from 1 January 1987) and outpatient visits (from 1 January 2001) in Sweden [13]. Data include birth date (month and year), sex, dates of admission and discharge from hospital and outpatient visits, birth country, and diagnoses according to the International Classification of Diseases, Tenth Revision (ICD‐10). In a recent validation study, 92.5% of MS diagnoses recorded in the patient register were confirmed in other Swedish registers [14].

First implemented in 2000, the MS registry collates clinical information from all 64 neurology clinics across Sweden and is estimated to capture 87% of all MS cases nationwide [15, 16, 17]. Data are recorded prospectively at each visit, and MS is diagnosed by a neurologist. In 2019, the MS registry was validated through a comprehensive medical chart review [18]. Variables retrieved from the MS registry for the current study were birth date (month and year), sex, and date of MS onset, as recorded by the treating neurologist.

Sex‐ and age‐specific data on the Swedish population were obtained from Statistics Sweden [19]. The world standard population, a composite developed in 1960 based on populations from 46 countries, was obtained from the National Cancer Institute [20]. Migration and death data for the MS cases were obtained from Statistics Sweden [19].

### Participants

MS cases were identified from the patient register using a validated algorithm requiring at least three MS‐specific ICD‐10 codes (G35) from inpatient or outpatient care occurring on different dates [21]. The date of the first central nervous system demyelinating diagnosis (ICD‐10: MS [G35], other acute disseminated demyelination [G36], neuromyelitis optica [G360], acute disseminated encephalomyelitis [G369], acute transverse myelitis [G373], demyelinating disease of the central nervous system (CNS) specified [G378], demyelinating disease of the CNS unspecified [G379], optic neuritis [H46]) was defined as the index date. An index date before age 18 years was considered a pediatric onset. MS cases identified in the MS registry with disease onset before age 18 years were also included as PoMS cases. For cases identified through both registers, the earliest of the two dates (MS onset in the MS registry or first demyelinating code in the patient register) was chosen as the index date.

For patients identified through the patient register, a 5‐year run‐in period with no CNS demyelinating disease codes before the index date was required for the incident cases. Therefore, cases had to be residents in Sweden for at least 5 years before the index date. Patients identified through the MS registry had to be residents in Sweden at the index date.

For the prevalence analyses, cases were included in the years they were residents in Sweden and meeting PoMS criteria until the month before their 18th birthday, emigration, or death.

### Standard protocol approvals, registrations, and patient consents

The study was approved by the Stockholm Regional Ethical Review Board (case no. 2017/1378‐31). All individual data from the different sources were made anonymous to the authors by replacing the personal identification numbers with study‐specific identifiers. Thus, informed consent was not required.

### Measurement and statistical methods

Crude incidence rates were estimated annually and during the entire study period for the total population and stratified by age group and sex, with 95% confidence intervals (CIs) assuming a Poisson distribution. The model offset was the total person‐time at risk for PoMS among children and youth younger than 18 years in the total population or respective strata. Temporal trends in the incidence rate and incidence rate ratios between sexes and age groups (with the middle age category as the reference group) were estimated using Poisson regression or negative binomial regression, depending on the data distribution. Unadjusted and index year‐, sex‐, and age group‐adjusted rate ratios with 95% CIs were reported. Furthermore, age‐ and sex‐standardized incidence rates were calculated using the direct method, with 95% CIs assuming a γ distribution.

Crude point prevalence was estimated annually and during the entire study period for the total population and stratified by age group and sex, with 95% CIs assuming a binomial distribution. The model offset was the population of children and youth younger than 18 years in the total population or respective strata. Unadjusted and sex‐ and age group‐adjusted estimates of temporal trends with 95% CIs were calculated using log binomial regression. Finally, age‐ and sex‐standardized prevalence estimates were calculated using the direct method, with 95% CIs assuming a γ distribution.

We report incidence rate and prevalence estimates from 1 January 2006 (to allow a 5‐year run‐in period from when all data sources became available in 2001) to 31 December 2016 (to compensate for the delayed identification of MS from the registers).

Continuous variables were summarized using mean (standard deviation) or median (range or interquartile range [IQR]) as appropriate, depending on the data distribution. Age was categorized into groups (<12, 12–15, 16–17 years), and calendar year was analyzed as a continuous variable. For the standardization, the world standard population [20] was used.

The significance level was set to α = 0.05. Analyses were conducted in R. The R package ggplot2 was used for the graphical presentation of the data.

## RESULTS

### Characteristics of the cohort

From 1 January 2006 to 31 December 2016, we identified 238 unique individuals with PoMS in Sweden. Among these persons, 174 were identified in the patient register and 222 in the MS registry, whereas 162 were identified through both registers. For those identified through both registers, the index date was a median (IQR) of 87 (17–240) days earlier in the MS registry than the patient register. Among the full cohort, there was a female predisposition (69%), and the median (range) age at onset (index date) was 16.4 (5.8–18.0) years. Five percent had an onset before age 12 years, and most (88%) were born in Sweden (Table 1).

**TABLE 1:** Characteristics of the cohort of incident cases of pediatric‐onset MS in Sweden, 2006–2016.

Characteristic	National patient register (*N* = 174)	Swedish MS registry (*N* = 222)	Total (*N* = 238)
Sex			
Male	58 (33.3%)	66 (29.7%)	73 (30.7%)
Female	116 (66.7%)	156 (70.3%)	165 (69.3%)
Age at index date (years)			
Mean (SD)	16.2 (1.73)	15.7 (2.02)	15.8 (1.99)
Median [minimum, maximum]	16.7 [8.21, 18.0]	16.3 [5.78, 17.9]	16.4 [5.78, 18.0]
Age group at index date			
<12 years	5 (2.9%)	12 (5.4%)	12 (5.0%)
12–15 years	58 (33.3%)	86 (38.7%)	91 (38.2%)
16–17 years	111 (63.8%)	124 (55.9%)	135 (56.7%)
Country/continent of birth			
Sweden	163 (93.7%)	194 (87.4%)	210 (88.2%)
Europe (excluding Sweden)	5 (2.9%)	13 (5.9%)	13 (5.5%)
Other	6 (3.4%)	15 (6.8%)	15 (6.3%)

*Note*: The Total column represents the entire cohort, including patients from both the patient register and the MS registry. For cases identified through both registers, the earliest of the two dates (MS onset in the MS registry or first demyelinating code in the patient register) was chosen as the index date. Abbreviation: MS, multiple sclerosis.

### Incidence rate

The overall crude incidence rate of PoMS was 1.12 (95% CI=0.98–1.27) per 100,000 person‐years, and the age‐ and sex‐standardized incidence rate was 1.07 (95% CI=0.94–1.22) per 100,000 person‐years over the study period (Table S1).

**FIGURE 1:**
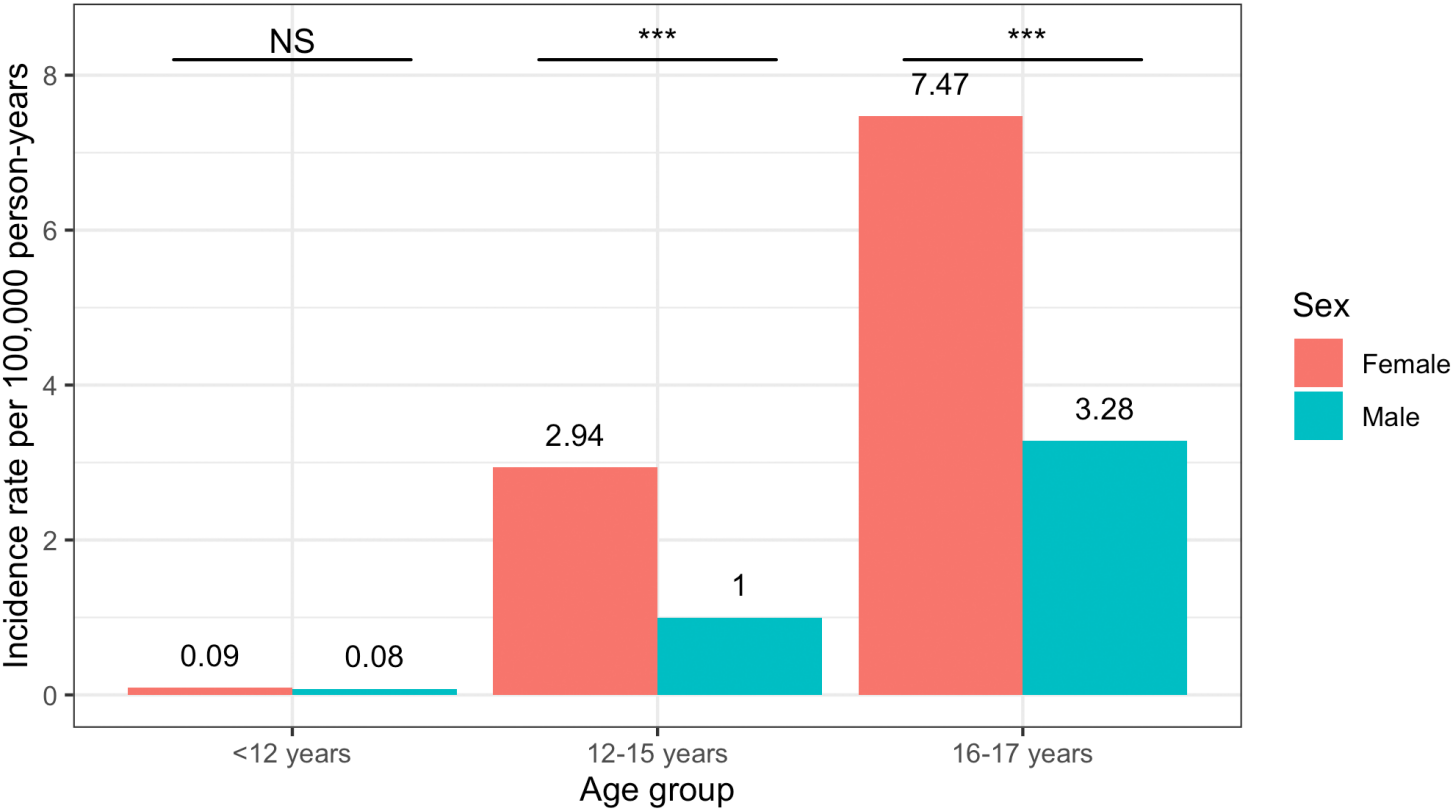
Sex‐specific incidence rates of pediatric‐onset multiple sclerosis in Sweden, 2006–2016, by age group. ***p < 0.001. NS, not significant.

The age group‐stratified incidence rates were 0.09 (95% CI=0.04–0.15) per 100,000 person‐years in the under 12 years old group, 1.94 (95% CI=1.56–2.38) per 100,000 person‐years in the 12–15 years old group, and 5.31 (95% CI=4.45–6.28) per 100,000 person‐years in the 16–17 years old group. Compared to the 12–15 years old group, the incidence rate was significantly higher (ratio 2.73; 95% CI=2.10–3.57) in the 16–17 years old group, whereas it was lower (ratio 0.04; 95% CI=0.02–0.08) in the under 12 years old group. Also, there was a significantly higher incidence rate among females than males, with a ratio of 2.40 (95% CI=1.83–3.18) (Table 2 and Table S2). When stratified by age group, there was a significantly higher preponderance of females in the two older age groups but not the youngest (Figure 1).

**TABLE 2:** Crude unadjusted and adjusted incidence rate ratios of pediatric‐onset multiple sclerosis in Sweden, 2006–2016.

Characteristic	Unadjusted			Adjusted^a^		
	IRR	95% CI	*p* value	IRR	95% CI	*p* value
Year (continuous)	0.96	0.91, 1.00	0.074	0.98	0.94, 1.02	0.4
Sex						
Male (reference)	—	—		—	—	
Female	2.39	1.82, 3.16	<0.001	2.40	1.83, 3.18	<0.001
Age group						
<12 years	0.04	0.02, 0.08	<0.001	0.04	0.02, 0.08	<0.001
12–15 years (reference)	—	—		—	—	
16–17 years	2.73	2.10, 3.57	<0.001	2.73	2.10, 3.57	<0.001

Abbreviations: CI, confidence interval; IRR, incidence rate ratio.

^a^ Adjusted for year, age group, and sex.

**FIGURE 2:**
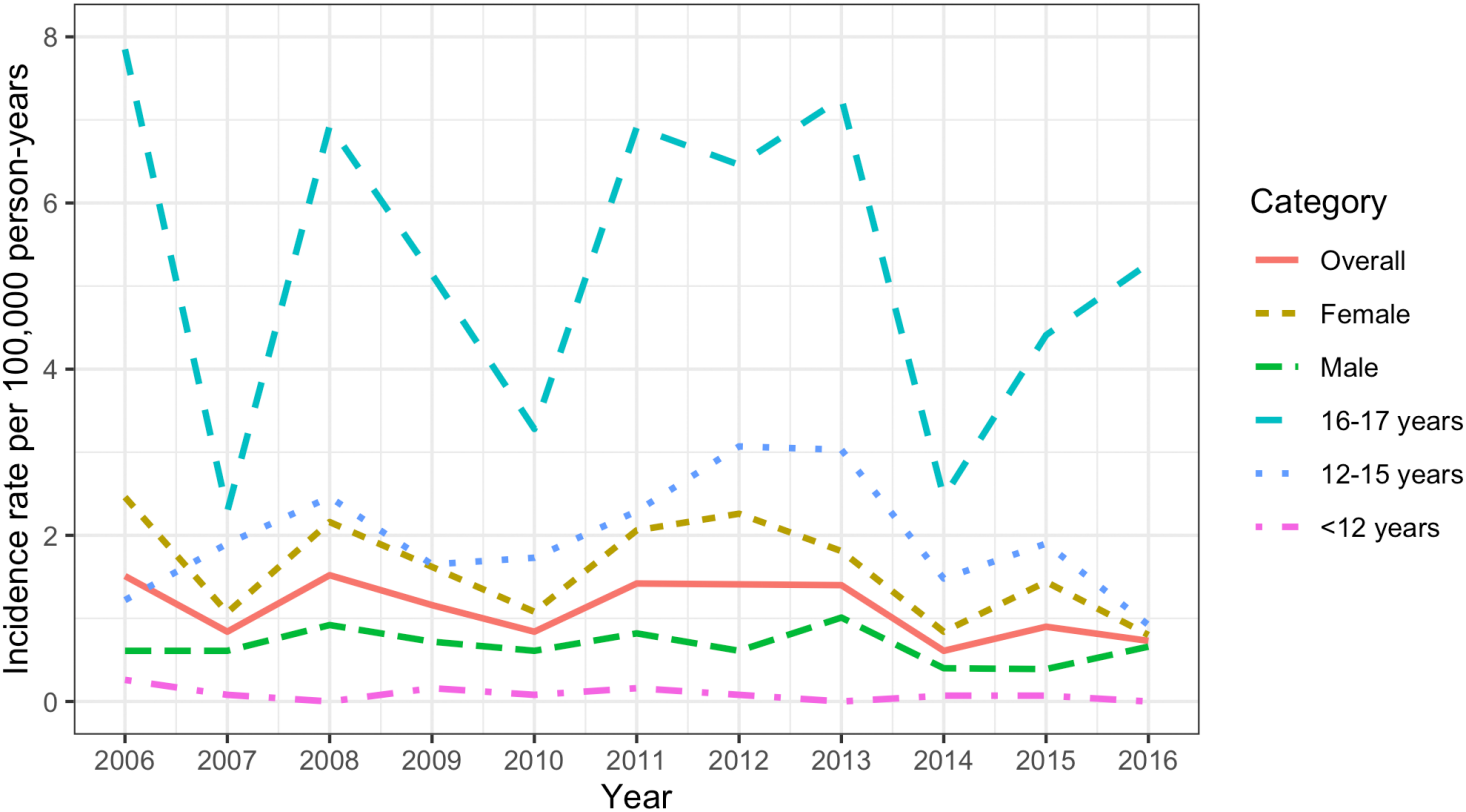
Crude incidence rates of pediatric‐onset multiple sclerosis in Sweden, 2006–2016, annually and stratified by sex and age group. The solid red line depicts the overall incidence rate, and the dashed lines represent the different strata. The annual incidence rates did not significantly change over time in the regression models.

The incidence rates did not significantly change over time for the total population or any of the strata (Figure 2 and Table S5).

### Prevalence

The overall crude prevalence was 2.82 (95% CI=2.60–3.06) per 100,000, and the overall age‐ and sex‐standardized prevalence was 2.69 (95% CI=2.48–2.92) per 100,000 (Table S3). The overall crude female‐to‐male prevalence ratio was 2.07 (95% CI=1.75–2.46, Table S4).

**FIGURE 3:**
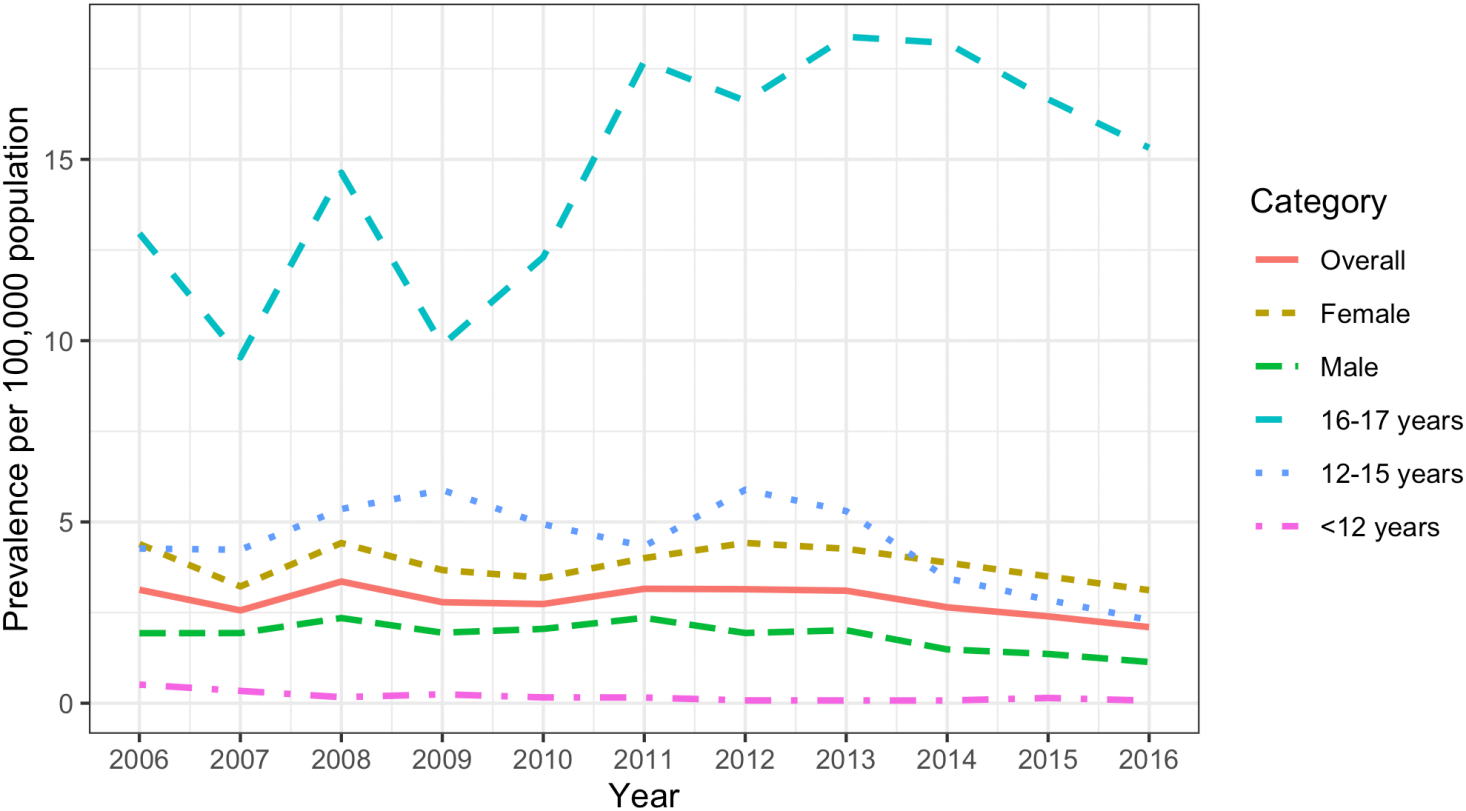
Crude annual prevalence of pediatric‐onset multiple sclerosis in Sweden, 2006–2016, overall and stratified by sex and age group. The solid red line depicts the overall prevalence, and the dashed lines represent the different strata. When adjusted for age group and/or sex, there was an annual 18% decrease among children under 12 years old (risk ratio 0.82, 95% CI=0.72–0.94), whereas there was an annual 5% increase among children 16–17 years old (1.05, 95% CI=1.01–1.08). There were no significant changes over time in the overall population.

There was no evidence of a temporal trend in the overall PoMS population. There was a decreasing trend in males (risk ratio 0.96; 95% CI=0.92–1.00) as well as in children <12 and 12–15 years old (risk ratios 0.82; 95% CI=0.72–0.94, and 0.96; 95% CI=0.92–1.00, respectively), whereas there was an annual 5% increase in prevalence (risk ratio 1.05; 95% CI=1.01–1.08) in children 16–17 years old. When adjusted for age group and/or sex, there were only significant trends among children <12 and 16–17 years old (risk ratios 0.82; 95% CI=0.72–0.94, and 1.05; 95% CI=1.01–1.08, respectively, Figure 3 and Table S6).

## DISCUSSION

In this nationwide registry‐based study of the incidence and prevalence of PoMS in Sweden, we identified 238 incident cases from 2006 to 2016. This corresponds to an overall crude incidence rate of 1.12 (95% CI=0.98–1.27) per 100,000 person‐years. The overall crude prevalence was 2.82 (95% CI=2.60–3.06) per 100,000 during the same period.

Studies of incidence rates that employed a similar methodology as ours (PoMS defined as onset at under 18 years old) have been performed in the Netherlands (89 cases, incidence rate 0.26 [95% CI=0.21–0.32]) [22] and Denmark (364 cases, incidence rate 0.79 [95% CI=0.71–0.88]) [23]. A recent Canadian population‐based study with data from British Columbia and Ontario included cases with an onset before 19 years old and reported average age‐standardized incidence rates of 0.95 (95% CI=0.79–1.13) and 0.98 (95% CI=0.84–1.12) [24]. Thus, in comparison, our estimates are high. There are other incidence studies, but few are population‐based, and the methodology varies widely regarding the upper age limit considered pediatric (16–20 years). Furthermore, some studies include patients based on age at first central demyelinating event, whereas some include patients based on age at MS diagnosis [8]. Although we did not have the ability to estimate the proportion of PoMS in the total Swedish MS population, a previous Swedish MS registry‐based cohort study of long‐term disability progression in MS reported a proportion of 4.4% [25], which aligns with the wider literature [26].

Our age‐stratified incidence rates were 0.09 per 100,000 person‐years in the under 12 years old group, 1.94 per 100,000 person‐years in the 12–15 years old group, and 5.31 per 100,000 person‐years in the 16–17 years old group. The under 12 years old group only consisted of 12 patients, so conclusions should be drawn carefully. Furthermore, due to privacy regulations, we cannot report on small cell sizes (<6), which limits our ability to provide details on this group. In the Danish study mentioned above, the incidence rate was 0.12 per 100,000 person‐years in the under 13 years old age group and 2.5 per 100,000 person‐years in the age group 12–17 years old [23]. In a German nationwide survey including patients with onset at under 16 years old, the under 11 years old group had an incidence rate of 0.09 per 100,000 person‐years, whereas it was 2.6 per 100,000 person‐years in the 14–15 years old age group [27]. Because the age categories and study periods (the Danish study dates back to 1977) are different across these studies, making comparisons is challenging. However, the incidence rate of PoMS in Sweden among younger children seems to be in alignment with other European countries, whereas the incidence rate among older children and adolescents is in the higher range of those previously reported.

There are few studies on the prevalence of PoMS. The Canadian study mentioned above reported increasing age‐standardized prevalence estimates from 4.75 (95% CI=3.44–6.40) to 5.52 (95% CI=4.11–7.27) in British Columbia and 2.93 (95% CI=2.45–3.47) to 4.07 (95% CI=3.39–4.85) in Ontario (reaching significance in Ontario only) during the period 2003–2019 [24]. The upper age limit of 19 likely contributed to the higher estimates in Canada. The wide‐ranging methodologies employed in previous PoMS prevalence studies, along with the limited number of cases in some of these studies, introduce substantial variability and complicate the prospect of conducting a meaningful comparison [8].

In alignment with the literature, we found an increasing incidence rate with increasing age amongst PoMS, with the most cases occurring in the oldest age category (16–17 years old) [8]. There was also a higher incidence rate among females overall. However, in the youngest age category, the sex ratio was almost equal. The increase in female‐to‐male ratio with age has been identified elsewhere, but the biological explanation has not been identified [8]. Puberty in females has been suggested to enhance CNS autoimmune mechanisms, for instance, myelin‐reactive T‐cell responses, which may contribute to the rising incidence [28].

In our study, the incidence rate fluctuated throughout the study period, but there was no evidence of a temporal trend. When adjusted for age group and/or sex, there were trends for a decreasing prevalence among the youngest age group and an increasing prevalence among the oldest age group. The increasing prevalence in the oldest age group could be explained by an increased incidence rate (either in the oldest group or the younger). However, we did not find evidence of such trends. An increasing awareness of myelin‐oligodendrocyte glycoprotein antibody‐associated disease (MOGAD) [29], especially among young children who may previously have been diagnosed with MS, could, to some extent, explain the decrease in prevalence in the youngest age group. Although not reaching significance, there was also a tendency of a decreasing incidence rate in this age group. Due to the modest numbers, conclusions should be made carefully for this age group. The overall age group‐ and sex‐adjusted prevalence remained stable.

### Strengths and limitations

Sweden offers an ideal setting for performing epidemiological studies of this nature, with well‐established and validated high‐quality nationwide registers. We were able to access both administrative and clinical data sets to maximize our ability to identify cases. Each register has its own strengths and limitations. The MS registry contains detailed clinical information, including the date of disease onset; however, it is not truly population‐based, with an estimated 87% coverage of the Swedish MS population. In contrast, the patient register is population‐based but lacks clinical detail, such that the onset of MS must be estimated based on a person's use of health services. Although we applied a validated algorithm for identifying cases from the patient register, we may have underestimated the true incidence rate and prevalence due to the moderate sensitivity of the algorithm and some missingness in the raw data set.

It is also possible that false positives were included. In other words, persons were identified as having MS when they had MOGAD or acute demyelinating encephalomyelitis, both of which are CNS demyelinating disorders that occur more frequently in children. MOGAD was still not well described during the study period, and testing for myelin‐oligodendrocyte glycoprotein (MOG)‐antibodies was not available until the very end of it. It is possible that some of our cases would now have been diagnosed as MOGAD, especially among the youngest. A Canadian study analyzed the serum of 65 persons diagnosed with pediatric MS and found that 12 (18%) were MOG‐immunoglobulin G positive [30]. These MOG‐positive patients were much younger, on average, than our population, with a median age of onset of 9 years. Therefore, it is unlikely that this misclassification would drastically alter our estimates, but our inability to differentiate MS from MOGAD is a limitation.

Furthermore, the MS registry is not as widely used among child neurologists as by adult neurologists. Consequently, PoMS patients are often not registered until they reach adulthood (18 years) and see an adult neurologist who retrospectively records their MS onset date. The impact that this could have had is that persons diagnosed in later years may have been missed, as they had not yet reached adulthood and been entered into the MS registry. To ensure that we were not underestimating the number of cases due to this delay, we excluded the final 3 years of data (2017–2019). Still, some young patients diagnosed at the end of the study period may have been missed.

Our use of the two registers in concert is a strength of this article. Finally, despite the relatively large numbers for a study of PoMS, the youngest age group was very small, precluding any strong conclusions.

## CONCLUSION

Sweden has a high incidence rate and prevalence of MS, and our results suggest that this is also true of PoMS. Both incidence rate and prevalence remained relatively stable during the study period, with no evidence of increasing or decreasing trends over time. Our results are valuable not only for the Swedish health care system and economy but also for the planning and implementation of clinical trials for PoMS, which are urgently needed for this vulnerable patient group.

## AUTHOR CONTRIBUTIONS

**Fredrik Sandesjö:** Conceptualization; methodology; formal analysis; writing – original draft; funding acquisition; investigation; visualization; writing – review and editing; project administration. **Helen Tremlett:** Conceptualization; methodology; writing – original draft; supervision; writing – review and editing. **Katarina Fink:** Conceptualization; writing – original draft; methodology; writing – review and editing; supervision. **Ruth Ann Marrie:** Conceptualization; writing – original draft; methodology; writing – review and editing; supervision. **Feng Zhu:** Conceptualization; writing – original draft; methodology; writing – review and editing; supervision; formal analysis. **Ronny Wickström:** Conceptualization; writing – original draft; funding acquisition; methodology; writing – review and editing; supervision. **Kyla A McKay:** Conceptualization; writing – original draft; funding acquisition; methodology; visualization; writing – review and editing; formal analysis; supervision.

## FUNDING INFORMATION

Financial support without any role in the design and conduct of this study was obtained from the Swedish Research Council for Health, Working Life and Welfare, and Sällskapet Barnavård.

## CONFLICT OF INTEREST STATEMENT

F.S. reports no disclosures relevant to the article. H.T. has, in the last 5 years, received research support from the Canada Research Chair Program, the National Multiple Sclerosis Society, the Canadian Institutes of Health Research, the Multiple Sclerosis Society of Canada, the Multiple Sclerosis Scientific Research Foundation, and the EDMUS Foundation (Fondation EDMUS contre la sclérose en plaques). In addition, in the last 5 years, H.T. has had travel expenses or registration fees prepaid or reimbursed to present at CME conferences from the Consortium of MS Centers (2018, 2023), National MS Society (2018, 2022), ECTRIMS/ACTRIMS (2017–2023), American Academy of Neurology (2019). Speaker honoraria are either declined or donated to an MS charity or to an unrestricted grant for use by H.T.'s research group. K.F. has received payment for lectures and advisory boards from Merck, Biogen, Novartis, Takeda, Roche, and Johnsson. R.A.M. receives research funding from CIHR, Research Manitoba, MS Canada, Crohn's and Colitis Canada, National Multiple Sclerosis Society, CMSC, the Arthritis Society, and the US Department of Defense, and is a coinvestigator on studies receiving funding from Biogen Idec and Roche Canada. F.Z. reports no disclosures relevant to the article. R.W. has received honoraria for serving on advisory boards for EISAI and Octapharma and speaker's fees from EISAI and Sanofi‐Genzyme. He has received funding from Region Stockholm Clinical Research Appointment. K.A.M. receives research funding support from the Swedish Research Council for Health, Working Life, and Welfare and StratNeuro, and has received speaker honoraria from Sanofi‐Aventis and Biogen.

## ETHICS STATEMENT

Ethical approval was granted by the Swedish Ethical Review Authority.

## Supporting information


Table S1.

Table S2.

Table S3.

Table S4.

Table S5.

Table S6.

## Data Availability

The data that support the findings of this study are available on request from the corresponding author. The data are not publicly available due to privacy or ethical restrictions.

## References

[1] Compston A , Coles A . Multiple sclerosis. Lancet. 2008;372(9648):1502‐1517. doi:10.1016/S0140-6736(08)61620-7 18970977

[2] Walton C , King R , Rechtman L , et al. Rising prevalence of multiple sclerosis worldwide: insights from the atlas of MS, third edition. Mult Scler J. 2020;26(14):1816‐1821. doi:10.1177/1352458520970841 PMC7720355 33174475

[3] Renoux C , Vukusic S , Mikaeloff Y , et al. Natural history of multiple sclerosis with childhood onset. N Engl J Med. 2007;356(25):2603‐2613. doi:10.1056/nejmoa067597 17582070

[4] Simone IL , Carrara D , Tortorella C , et al. Course and prognosis in early‐onset MS: comparison with adult‐onset forms. Neurology. 2002;59(12):1922‐1928. doi:10.1212/01.wnl.0000036907.37650.8e 12499484

[5] Gorman MP , Healy BC , Polgar‐Turcsanyi M , Chitnis T . Increased relapse rate in pediatric‐onset compared with adult‐onset multiple sclerosis. Arch Neurol. 2009;66(1):54‐59. doi:10.1001/archneurol.2008.505 19139299

[6] Waubant E , Chabas D , Okuda DT , et al. Difference in disease burden and activity in pediatric patients on brain magnetic resonance imaging at time of multiple sclerosis onset vs adults. Arch Neurol. 2009;66(8):967‐971. doi:10.1001/archneurol.2009.135 19667217

[7] Amato MP , Krupp LB , Charvet LE , Penner I , Till C . Pediatric multiple sclerosis. Neurology. 2016;87(9 Supplement 2):S82‐S87. doi:10.1212/wnl.0000000000002883 27572867

[8] Jeong A , Oleske DM , Holman J . Epidemiology of pediatric‐onset multiple sclerosis: a systematic review of the literature. J Child Neurol. 2019;34(12):705‐712. doi:10.1177/0883073819845827 31185780

[9] Ahlgren C , Odén A , Lycke J . High nationwide prevalence of multiple sclerosis in Sweden. Mult Scler J. 2011;17(8):901‐908. doi:10.1177/1352458511403794 21459810

[10] Ahlgren C , Odén A , Lycke J . High Nationwide incidence of multiple sclerosis in Sweden. PLoS ONE. 2014;9(9):e108599. doi:10.1371/journal.pone.0108599 25265372 PMC4180935

[11] About the Swedish healthcare system. Socialstyrelsen. Updated September 01, 2020. Accessed September 21, 2023. https://www.socialstyrelsen.se/en/about‐us/healthcare‐for‐visitors‐to‐sweden/about‐the‐swedish‐healthcare‐system/

[12] Population in Sweden by Country/Region of Birth, Citizenship and Swedish/Foreign background, 31 December 2022. Statistics Sweden. Updated March 22, 2023. Accessed October 4, 2023. https://www.scb.se/en/finding‐statistics/statistics‐by‐subject‐area/population/population‐composition/population‐statistics/pong/tables‐and‐graphs/foreign‐born‐citizenship‐and‐foreignswedish‐background/population‐in‐sweden‐by‐countryregion‐of‐birth‐citizenship‐and‐swedishforeign‐background‐31‐december‐2022/

[13] The National Patient Register. Socialstyrelsen Updated January 14, 2022. Accessed September 28, 2023. https://www.socialstyrelsen.se/en/statistics‐and‐data/registers/national‐patient‐register/

[14] Murley C , Friberg E , Hillert J , Alexanderson K , Yang F . Validation of multiple sclerosis diagnoses in the Swedish National Patient Register. Eur J Epidemiol. 2019;34(12):1161‐1169. doi:10.1007/s10654-019-00558-7 31493189 PMC7010617

[15] Andersen O . From the Gothenburg cohort to the Swedish multiple sclerosis registry. Acta Neurol Scand. 2012;126:13‐19. doi:10.1111/ane.12023 23278651

[16] Hillert J , Stawiarz L . The Swedish MS registry – clinical support tool and scientific resource. Acta Neurol Scand. 2015;132(S199):11‐19. doi:10.1111/ane.12425 26046553 PMC4657484

[17] Årsrapport för 2022 Multipel Skleros Swedish neuro registers. Accessed October, 9, 2023. https://www.neuroreg.se/media/atojdkq1/årsrapport‐för‐2022_multipel‐skleros.pdf

[18] Alping P , Piehl F , Langer‐Gould A , Frisell T . Validation of the Swedish multiple sclerosis register. Epidemiology. 2019;30(2):230‐233. doi:10.1097/ede.0000000000000948 30721167 PMC6369893

[19] Population by age and sex. Year 1860–2022. Statistics Sweden. Updated February 22, 2023. Accessed February 20, 2022. https://www.statistikdatabasen.scb.se/pxweb/en/ssd/START__BE__BE0101__BE0101A/BefolkningR1860N/

[20] Standard Populations (Millions) for Age‐Adjustment. National Cancer Institute. Accessed December 06, 2022. https://seer.cancer.gov/stdpopulations/

[21] Teljas C , Boström I , Marrie RA , et al. Validating the diagnosis of multiple sclerosis using Swedish administrative data in Värmland County. Acta Neurol Scand. 2021;144(6):680‐686. doi:10.1111/ane.13514 34357597

[22] De Mol CL , Wong YYM , Van Pelt ED , et al. Incidence and outcome of acquired demyelinating syndromes in Dutch children: update of a nationwide and prospective study. J Neurol. 2018;265(6):1310‐1319. doi:10.1007/s00415-018-8835-6 29569176 PMC5990581

[23] Boesen MS , Magyari M , Koch‐Henriksen N , et al. Pediatric‐onset multiple sclerosis and other acquired demyelinating syndromes of the central nervous system in Denmark during 1977–2015: a nationwide population‐based incidence study. Mult Scler J. 2018;24(8):1077‐1086. doi:10.1177/1352458517713669 28608742

[24] Yusuf FLA , Asaf A , Marrie RA , et al. Incidence and prevalence of paediatric‐onset multiple sclerosis in two Canadian provinces: a population‐based study representing over half of Canada's population. J Neurol Neurosurg Psychiatry. 2024;95(3):229‐234. doi:10.1136/jnnp-2023-331991 37734925

[25] Mckay KA , Hillert J , Manouchehrinia A . Long‐term disability progression of pediatric‐onset multiple sclerosis. Neurology. 2019;92(24):e2764‐e2773. doi:10.1212/wnl.0000000000007647 31092624 PMC6598792

[26] Chabas D , Green AJ , Waubant E . Pediatric multiple sclerosis. NeuroRx. 2006;3(2):264‐275. doi:10.1016/j.nurx.2006.01.011 16554264 PMC3593440

[27] Reinhardt K , Weiss S , Rosenbauer J , Gartner J , von Kries R . Multiple sclerosis in children and adolescents: incidence and clinical picture – new insights from the nationwide German surveillance (2009–2011). Eur J Neurol. 2014;21(4):654‐659. doi:10.1111/ene.12371 24471864

[28] Ahn JJ , O'Mahony J , Moshkova M , et al. Puberty in females enhances the risk of an outcome of multiple sclerosis in children and the development of central nervous system autoimmunity in mice. Mult Scler. 2015;21(6):735‐748. doi:10.1177/1352458514551453 25533291

[29] Fadda G , Armangue T , Hacohen Y , Chitnis T , Banwell B . Paediatric multiple sclerosis and antibody‐associated demyelination: clinical, imaging, and biological considerations for diagnosis and care. Lancet Neurol. 2021;20(2):136‐149. doi:10.1016/s1474-4422(20)30432-4 33484648

[30] Fadda G , Waters P , Woodhall M , et al. Serum MOG‐IgG in children meeting multiple sclerosis diagnostic criteria. Mult Scler J. 2022;28(11):1697‐1709. doi:10.1177/13524585221093789 PMC9442635 35581944

