# Supplementary material for: Incidence rate and prevalence of pediatric‐onset multiple sclerosis in Sweden: A population‐based register study
Source: Eur J Neurol. 2024 Feb 18;31(5):e16253. doi: 10.1111/ene.16253 (PMC11236061; doi:10.1111/ene.16253)
Supplement: Supplementary file 1 — Table S1. [file ENE-31-e16253-s003.docx]

**eTable 1.** Annual and overall crude and age- and sex-standardized incidence rates of pediatric-onset multiple sclerosis per 100,000 person-years in the population aged <18 years in Sweden, 2006 – 2016.

| **Year** | **Cases** | **Person-years at risk** | **Crude incidence** | **95% CI** | **Age- and sex-standardized incidence** | **95% CI** |
| --- | --- | --- | --- | --- | --- | --- |
| 2006 | 29 | 1 917 127 | 1.51 | 1.01-2.17 | 1.31 | 0.88-1.91 |
| 2007 | 16 | 1 913 372 | 0.84 | 0.48-1.36 | 0.73 | 0.42-1.21 |
| 2008 | 29 | 1 906 876 | 1.52 | 1.02-2.18 | 1.31 | 0.87-1.90 |
| 2009 | 22 | 1 902 483 | 1.16 | 0.72-1.75 | 1.04 | 0.65-1.59 |
| 2010 | 16 | 1 900 742 | 0.84 | 0.48-1.37 | 0.79 | 0.45-1.30 |
| 2011 | 27 | 1 901 223 | 1.42 | 0.94-2.07 | 1.38 | 0.91-2.01 |
| 2012 | 27 | 1 908 246 | 1.41 | 0.93-2.06 | 1.45 | 0.96-2.12 |
| 2013 | 27 | 1 932 881 | 1.40 | 0.92-2.03 | 1.47 | 0.97-2.14 |
| 2014 | 12 | 1 964 560 | 0.61 | 0.32-1.07 | 0.65 | 0.33-1.14 |
| 2015 | 18 | 2 003 835 | 0.90 | 0.53-1.42 | 0.96 | 0.57-1.52 |
| 2016 | 15 | 2 050 006 | 0.73 | 0.41-1.21 | 0.78 | 0.43-1.28 |
| Overall | 238 | 21 301 349 | 1.12 | 0.98-1.27 | 1.07 | 0.94-1.22 |

CI, Confidence Interval
