# Supplementary material for: Incidence rate and prevalence of pediatric‐onset multiple sclerosis in Sweden: A population‐based register study
Source: Eur J Neurol. 2024 Feb 18;31(5):e16253. doi: 10.1111/ene.16253 (PMC11236061; doi:10.1111/ene.16253)
Supplement: Supplementary file 2 — Table S2. [file ENE-31-e16253-s005.docx]

**eTable 2.** Annual and overall crude and age-standardized incidence rates of pediatric-onset multiple sclerosis per 100,000 person-years in the population aged <18 years in Sweden, 2006 – 2016, stratified by sex. Incidence rate ratios are crude.

|  | **Females** | | | | **Males** | | | | **IRR - F:M** | |
| --- | --- | --- | --- | --- | --- | --- | --- | --- | --- | --- |
|  | **Crude** | | **Age standardized** | | **Crude** | | **Age standardized** | |  |  |
| **Year** | **IR** | **95% CI** | **IR** | **95% CI** | **IR** | **95% CI** | **IR** | **95% CI** | **IRR** | **95% CI** |
| 2006 | 2.46 | 1.56-3.70 | 2.09 | 1.32-3.19 | 0.61 | 0.22-1.33 | 0.54 | 0.19-1.21 | 4.03 | 1.75-10.9 |
| 2007 | 1.07 | 0.51-1.97 | 0.91 | 0.44-1.73 | 0.61 | 0.22-1.33 | 0.54 | 0.20-1.23 | 1.75 | 0.65-5.16 |
| 2008 | 2.16 | 1.32-3.33 | 1.84 | 1.12-2.88 | 0.92 | 0.42-1.75 | 0.77 | 0.35-1.51 | 2.35 | 1.10-5.42 |
| 2009 | 1.62 | 0.91-2.67 | 1.44 | 0.80-2.40 | 0.72 | 0.29-1.48 | 0.64 | 0.26-1.36 | 2.25 | 0.95-5.92 |
| 2010 | 1.08 | 0.52-1.99 | 0.97 | 0.47-1.82 | 0.61 | 0.23-1.34 | 0.61 | 0.22-1.34 | 1.77 | 0.65-5.17 |
| 2011 | 2.06 | 1.24-3.21 | 1.98 | 1.19-3.11 | 0.82 | 0.35-1.61 | 0.78 | 0.33-1.55 | 2.51 | 1.14-6.08 |
| 2012 | 2.26 | 1.40-3.46 | 2.30 | 1.42-3.52 | 0.61 | 0.22-1.33 | 0.61 | 0.22-1.34 | 3.70 | 1.59-10.1 |
| 2013 | 1.81 | 1.05-2.90 | 1.90 | 1.10-3.04 | 1.01 | 0.48-1.85 | 1.04 | 0.50-1.91 | 1.79 | 0.84-4.08 |
| 2014 | 0.84 | 0.36-1.65 | 0.88 | 0.38-1.74 | 0.40 | 0.11-1.01 | 0.42 | 0.11-1.07 | 2.10 | 0.67-7.94 |
| 2015 | 1.44 | 0.79-2.42 | 1.51 | 0.83-2.54 | 0.39 | 0.11-0.99 | 0.41 | 0.11-1.05 | 3.69 | 1.33-13.1 |
| 2016 | 0.80 | 0.35-1.59 | 0.85 | 0.37-1.68 | 0.66 | 0.27-1.37 | 0.70 | 0.28-1.44 | 1.21 | 0.44-3.46 |
| Overall | 1.59 | 1.36-1.86 | 1.52 | 1.29-1.77 | 0.67 | 0.52-0.84 | 0.63 | 0.49-0.79 | 2.39 | 1.82-3.16 |

IR, Incidence Rate; IRR, Incidence Rate Ratio; CI, Confidence Interval; F:M, Female to male
