# Supplementary material for: Incidence rate and prevalence of pediatric‐onset multiple sclerosis in Sweden: A population‐based register study
Source: Eur J Neurol. 2024 Feb 18;31(5):e16253. doi: 10.1111/ene.16253 (PMC11236061; doi:10.1111/ene.16253)
Supplement: Supplementary file 3 — Table S3. [file ENE-31-e16253-s001.docx]

**eTable 3.** Annual and overall crude and age- and sex-standardized prevalence of pediatric-onset multiple sclerosis per 100,000 people aged <18 years in Sweden, 2006 – 2018.

| **Year** | **Prevalent Cases** | **Population** | **Crude prevalence** | **95% CI** | **Age- and sex- standardized prevalence** | **95% CI** |
| --- | --- | --- | --- | --- | --- | --- |
| 2006 | 60 | 1 917 143 | 3.13 | 2.39-4.03 | 2.72 | 2.07-3.52 |
| 2007 | 49 | 1 913 439 | 2.56 | 1.89-3.39 | 2.21 | 1.63-2.94 |
| 2008 | 64 | 1 906 940 | 3.36 | 2.58-4.29 | 2.90 | 2.23-3.73 |
| 2009 | 53 | 1 902 558 | 2.79 | 2.09-3.64 | 2.55 | 1.91-3.35 |
| 2010 | 52 | 1 900 804 | 2.74 | 2.04-3.59 | 2.54 | 1.90-3.35 |
| 2011 | 60 | 1 901 291 | 3.16 | 2.41-4.06 | 3.00 | 2.29-3.88 |
| 2012 | 60 | 1 908 322 | 3.14 | 2.40-4.05 | 3.19 | 2.43-4.11 |
| 2013 | 60 | 1 932 957 | 3.10 | 2.37-4.00 | 3.25 | 2.48-4.19 |
| 2014 | 52 | 1 964 627 | 2.65 | 1.98-3.47 | 2.83 | 2.11-3.71 |
| 2015 | 48 | 2 003 895 | 2.40 | 1.77-3.18 | 2.57 | 1.89-3.41 |
| 2016 | 43 | 2 050 061 | 2.10 | 1.52-2.83 | 2.26 | 1.63-3.04 |
| Overall | 601 | 21 302 037 | 2.82 | 2.60-3.06 | 2.69 | 2.48-2.92 |

CI, Confidence Interval
