# Supplementary material for: Incidence rate and prevalence of pediatric‐onset multiple sclerosis in Sweden: A population‐based register study
Source: Eur J Neurol. 2024 Feb 18;31(5):e16253. doi: 10.1111/ene.16253 (PMC11236061; doi:10.1111/ene.16253)
Supplement: Supplementary file 4 — Table S4. [file ENE-31-e16253-s004.docx]

**eTable 4.**  Annual and overall crude and age-standardized prevalence of pediatric-onset multiple sclerosis per 100,000 people aged <18 years in Sweden 2006-2016, stratified by sex. Risk ratios are crude.

|  | **Female** | | | | **Males** | | | | **RR - F:M** | |
| --- | --- | --- | --- | --- | --- | --- | --- | --- | --- | --- |
| **Year** | **Crude prevalence** | **95% CI** | **Age- standardized prevalence** | **95% CI** | **Crude prevalence** | **95% CI** | **Age- standardized prevalence** | **95% CI** | **RR** | **95% CI** |
| 2006 | 4.39 | 3.15-5.96 | 3.77 | 2.70-5.16 | 1.93 | 1.16-3.02 | 1.66 | 1.00-2.64 | 2.27 | 1.34-4.00 |
| 2007 | 3.22 | 2.17-4.60 | 2.74 | 1.85-3.96 | 1.94 | 1.17-3.02 | 1.67 | 1.00-2.65 | 1.66 | 0.94-3.01 |
| 2008 | 4.42 | 3.17-5.99 | 3.77 | 2.70-5.15 | 2.35 | 1.49-3.52 | 2.04 | 1.29-3.10 | 1.88 | 1.14-3.18 |
| 2009 | 3.67 | 2.54-5.13 | 3.29 | 2.27-4.63 | 1.95 | 1.17-3.04 | 1.81 | 1.08-2.84 | 1.89 | 1.09-3.37 |
| 2010 | 3.46 | 2.37-4.88 | 3.17 | 2.16-4.49 | 2.05 | 1.25-3.16 | 1.92 | 1.17-2.99 | 1.69 | 0.97-3.00 |
| 2011 | 4.00 | 2.82-5.52 | 3.77 | 2.65-5.22 | 2.35 | 1.49-3.53 | 2.24 | 1.42-3.38 | 1.70 | 1.02-2.90 |
| 2012 | 4.42 | 3.17-6.00 | 4.46 | 3.20-6.06 | 1.94 | 1.17-3.02 | 1.92 | 1.16-3.01 | 2.28 | 1.34-4.02 |
| 2013 | 4.26 | 3.04-5.80 | 4.42 | 3.16-6.03 | 2.01 | 1.23-3.11 | 2.07 | 1.27-3.21 | 2.12 | 1.25-3.69 |
| 2014 | 3.88 | 2.73-5.34 | 4.11 | 2.90-5.67 | 1.48 | 0.83-2.45 | 1.55 | 0.87-2.55 | 2.61 | 1.46-4.91 |
| 2015 | 3.50 | 2.42-4.89 | 3.71 | 2.57-5.19 | 1.36 | 0.74-2.28 | 1.43 | 0.78-2.40 | 2.58 | 1.41-4.96 |
| 2016 | 3.12 | 2.12-4.43 | 3.31 | 2.25-4.71 | 1.14 | 0.59-1.99 | 1.20 | 0.62-2.09 | 2.74 | 1.45-5.56 |
| Overall | 3.84 | 3.47-4.24 | 3.63 | 3.29-4.01 | 1.85 | 1.61-2.13 | 1.75 | 1.52-2.01 | 2.07 | 1.75-2.46 |

CI, Confidence Interval; RR, Risk Ratio; F:M, Female to male
