# Supplementary material for: Incidence rate and prevalence of pediatric‐onset multiple sclerosis in Sweden: A population‐based register study
Source: Eur J Neurol. 2024 Feb 18;31(5):e16253. doi: 10.1111/ene.16253 (PMC11236061; doi:10.1111/ene.16253)
Supplement: Supplementary file 5 — Table S5. [file ENE-31-e16253-s006.docx]

**eTable 5.** Temporal trends in the incidence rate of pediatric-onset multiple sclerosis per 100,000 people aged <18 years in Sweden 2006-2016. Unadjusted and age and/or sex-adjusted estimates.

|  | **Unadjusted** | | | **Age- and/or sex adjusted** | | |
| --- | --- | --- | --- | --- | --- | --- |
| **Characteristic** | **IRR** | **95% CI** | **p-value** | **IRR** | **95% CI** | **p-value** |
| Overall | 0.96 | 0.91, 1.00 | 0.074 | 0.98 | 0.94, 1.02 | 0.4 |
| Females | 0.95 | 0.89, 1.01 | 0.074 | 0.97 | 0.92, 1.03 | 0.3 |
| Males | 0.98 | 0.91, 1.05 | 0.5 | 1.00 | 0.93, 1.08 | >0.9 |
| < 12 years | 0.85 | 0.70, 1.03 | 0.10 | 0.85 | 0.70, 1.03 | 0.10 |
| 12 - 15 years | 1.00 | 0.94, 1.06 | >0.9 | 1.00 | 0.94, 1.06 | >0.9 |
| 16 - 17 years | 0.98 | 0.92, 1.05 | 0.6 | 0.99 | 0.93, 1.05 | 0.7 |

IRR, Incidence Rate Ratio; CI, Confidence Interval.
